# Impaired holistic coding of facial expression and facial identity in congenital prosopagnosia

**DOI:** 10.1016/j.neuropsychologia.2011.02.021

**Published:** 2011-04

**Authors:** Romina Palermo, Megan L. Willis, Davide Rivolta, Elinor McKone, C. Ellie Wilson, Andrew J. Calder

**Affiliations:** a ARC Centre of Excellence in Cognition and its Disorders (CCD); b Department of Psychology, Australian National University, Canberra, ACT 0200, Australia; c Macquarie Centre for Cognitive Science (MACCS), Macquarie University, Sydney, NSW 2109, Australia; d MRC Cognition and Brain Sciences Unit, Cambridge CB2 7EF, England, United Kingdom

**Keywords:** Face perception, Identity, Expression, Emotion, Holistic processing, Prosopagnosia

## Abstract

We test 12 individuals with congenital prosopagnosia (CP), who replicate a common pattern of showing severe difficulty in recognising facial identity in conjunction with normal recognition of facial expressions (both basic and ‘social’). Strength of holistic processing was examined using standard expression composite and identity composite tasks. Compared to age- and sex-matched controls, group analyses demonstrated that CPs showed weaker holistic processing, for both expression and identity information. Implications are (a) normal expression recognition in CP can derive from compensatory strategies (e.g., over-reliance on non-holistic cues to expression); (b) the split between processing of expression and identity information may take place after a common stage of holistic processing; and (c) contrary to a recent claim, holistic processing of identity is functionally involved in face identification ability.

## Research highlights

► Congenital prosopagnosics show weak holistic coding of expression and identity. ► Normal expression recognition can result from compensatory strategies. ► There may be a common stage of holistic coding for expression and identity. ► Holistic coding of identity is functionally involved in face identification ability.

## 1 Introduction

People with congenital prosopagnosia (CP; also referred to as developmental prosopagnosia) have severe, life-long deficits recognising the identity of familiar people from their faces despite intact low-level vision and general cognitive abilities (Behrmann & Avidan, 2005; Lee, Duchaine, Wilson, & Nakayama, 2010). As many as 2.5% of the educated population can be classified as a CP (Bowles et al., 2009; Kennerknecht et al., 2006), and some of these cases run in families (Duchaine, Germine, & Nakayama, 2007; Grueter et al., 2007; Lee et al., 2010; Schmalzl, Palermo, & Coltheart, 2008). Face recognition deficits in CP appear to be associated with smaller anterior fusiform volumes (Behrmann, Avidan, Gao, & Black, 2007), reduced grey matter volume in brain regions that respond to faces, such as the mid-fusiform gyrus (Garrido et al., 2009), and compromised white matter tracts in occipito-temporal cortex (Thomas et al., 2009).

Despite profound impairments in facial identity recognition, many, although not all, CPs are adept at labelling the basic facial expressions of happiness, anger, disgust, fear, sadness and surprise (Kress & Daum, 2003; Nunn, Postma, & Pearson, 2001; Schmalzl et al., 2008), even when the display of these basic expressions is subtle and difficult to categorise (Duchaine, Parker, & Nakayama, 2003; Humphreys, Avidan, & Behrmann, 2007). CPs are also typically able to recognise subtle social emotions conveyed by the eyes, such as playfulness and regret (Duchaine et al., 2003, 2007; Lee et al., 2010). In the present study we test 12 CPs showing this pattern of impaired identity recognition with no discernable deficit in facial expression recognition, and examine the strength of holistic processing for face expression information (in all 12 participants) and face identity information (in a subset of nine participants), in order to address three theoretical questions regarding the role of holistic processing in their patterns of face processing abilities.

**Fig. 1:**
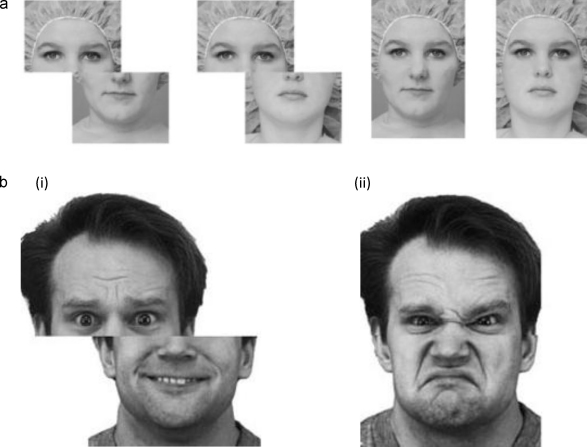
(a) *Identity composite task*. Pairs of faces were shown sequentially and participants judged whether the top halves of the faces were either the “same” or “different”. In this example the top halves were the “same” in both the unaligned (left) and aligned (right) pairs. The bottom halves were always of a different individual to the top individual. Reprinted with kind permission from Le Grand et al. (2004). (b) *Expression Composite task*. Examples of unaligned (left) and aligned (right) composite expressions in which participants judged the expression from either the (i) top (fear) or (ii) bottom (disgusted) halves of the face. The other half of the face was always of a different expression. Face images are from the KDEF (Lundqvist et al., 1998).

Holistic processing, defined as the “simultaneous perception of multiple features of an individual face, that are integrated into a single global representation” (Rossion, 2008, p. 275), is a core perceptual mechanism in the processing of faces. The most widely accepted measure of holistic processing is the composite effect. Assessing holistic coding of identity typically involves participants identifying the top (or bottom) half of a face paired with the bottom (or top) half of another person's face. The composite effect is the robust finding that participants are slower, and less accurate, when the face halves are vertically *aligned* (forming the illusion of a new face) compared to when they are spatially *unaligned* so they do not resemble a whole face (e.g., McKone, 2008; Young, Hellawell, & Hay, 1987; e.g., Fig. 1a). In the expression version, participants judge the expression on one half of the face (e.g., anger) while trying to ignore an inconsistent expression on the other half (e.g., happiness) (Calder & Jansen, 2005; Calder, Young, Keane, & Dean, 2000; White, 2000; e.g., Fig. 1b). Composite effects for both types of information occur for upright faces (where identity and expression recognition is typically also good) but not inverted faces (where recognition is poorer).

The first question we address in the current study is whether the normal levels of expression recognition ability in our CPs derive from normal use of perceptual mechanisms, as opposed to reliance on other compensatory strategies. Patient H.J.A., who acquired prosopagnosia at age 61 following a stroke, demonstrated a relatively normal ability to recognise facial expressions despite displaying no expression composite effect, implying the use of compensatory strategies (perhaps a reliance on local part cues) (Baudouin & Humphreys, 2006). This implies that there is no guarantee that normal expression recognition ability in CP is achieved via the same perceptual mechanisms used by typically developing adults. Here we test whether our CPs may rely less on holistic processing (and therefore more on other contributory mechanisms) than controls.

**Fig. 2:**
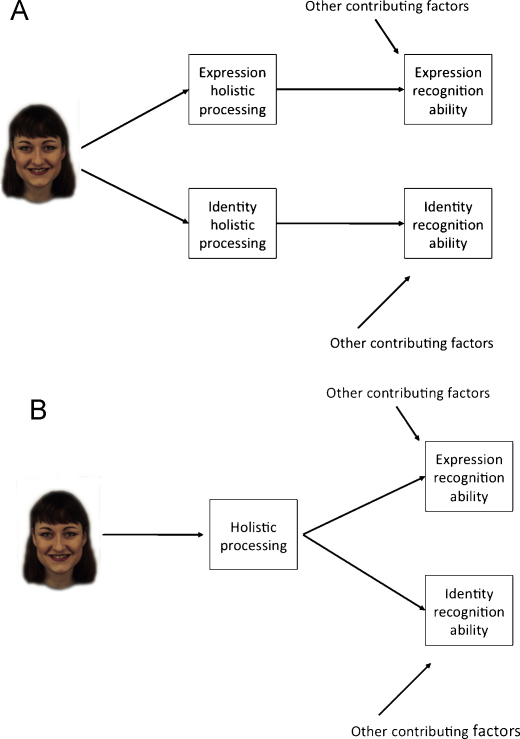
Models of holistic processing in identity and expression processing. In Model A, there are two separate holistic processing stages, one for expression and one for identity. In Model B, there is a holistic coding stage that is common to both expression and identity processing. Face image is from the KDEF (Lundqvist et al., 1998).

The second question concerns the stage of processing from which the dissociation between identity and expression recognition derives. Common theories place the point of split between processing of identity and expression information quite early in perceptual/cognitive processing, with the split occurring before the stage of view-independent ‘structural descriptions’ in the cognitive model of Bruce and Young (1986), and before processing in the lateral fusiform gyrus (identity) and superior temporal sulcus (expression) in the anatomical model of Haxby, Hoffman, and Gobbini (2000). However, Calder and Young's (2005) review argued that much of the evidence for an early split was not as strong as often assumed. Here, we address the question of whether a split has occurred by the perceptual stage of holistic processing. We consider two models. In the first (Model A, Fig. 2), there are two distinct types of holistic coding, one for coding expression and another for coding information about identity. In support of this model, Calder et al. (2000) found that participants could selectively attend to holistic information specific to identity or expression (i.e., participants took no longer to judge the facial expression of a composite whether they were composed of the same or different identity and vice versa), suggesting that holistic coding of identity and expression were independent. However, as this data can also be modelled within a single multi-dimensional system, there may not be an absolute dissociation between the composite effect for expression and identity (Calder and Young, 2005). Alternatively then, there could be a common stage of holistic coding, which feeds into both expression and identity recognition (Model B, Fig. 2). In support of this model, Calder and Jansen (2005) note that composite effects for both identity and expression are sensitive to inversion but not photographic negative, suggesting a common level of perceptual processing.

In the current study, we assess whether holistic expression and identity processing go together, or dissociate, in our CPs. Model A (two separate holistic processing stages) would be supported if a dissociation is revealed, that is, if one form of holistic processing is impaired, while the other is intact. Given that the CPs had impaired recognition of identity but not expression, such a dissociation would most likely take the form of the CPs showing a weak identity composite effect relative to controls but a normal strength expression composite effect. Alternatively, Model B (one combined holistic processing stage) would predict that the status of holistic processing of expression should match that of identity (i.e., either both impaired, or both intact). For example, if CPs showed weak composite effects for identity information, then Model B would also predict weak composite effects for expression information *despite* the CPs’ intact recognition of expressions.

The third question concerns identity processing only, and asks whether holistic processing of identity information is functionally related to face recognition ability. A recent paper with typically developing adults demonstrated large individual differences in the strength of the identity composite effect but found no correlation between these differences and face recognition ability (Konar, Bennett, & Sekuler, 2010). However, face recognition ability was assessed with simultaneous or immediate sequential matching tasks (where the stimuli included hair), and such tasks have been shown to be more closely associated with general object processing than face memory skills (e.g., the Glasgow Face Matching Task, GFMT; Burton, White, & McNeill, 2010). None of the CPs tested in our lab are impaired on the GFMT whereas they all show significant impairments on tests involving a memory component, such as the Cambridge Face Memory Test (CFMT) (unpublished data). In the current study we used our group-based comparison of differences in identity recognition ability (i.e., CPs vs. controls). If holistic processing is not functionally involved in recognition, then there should be no difference in the strength of the identity composite effect between the CP and control groups. On the other hand, if holistic coding contributes to face identity recognition, then the identity composite effect should be weaker in CPs than in controls.

To summarise, we confirmed impaired face identity with intact facial expression recognition in a group of 12 individuals. We then report the first group study of holistic coding of both expression and identity in CP.

## 2 Methods and results

### 2.1 Participants

#### 2.1.1 Congenital prosopagnosics

The CP group comprised 12 people (4 males) who reported severe everyday face recognition difficulties and performed poorly on tests of facial identity recognition. Most contacted us via our prosopagnosia register: http://www.maccs.mq.edu.au/research/projects/prosopagnosia/. They were aged between 20 and 60 years (*M* = 40.58, *SD* = 13.00) when facial identity recognition, low-level vision and IQ were assessed. They reported normal or corrected-to-normal vision and demonstrated normal range contrast sensitivity when measured with the *Functional Acuity Contrast Test* (FACT, Vision Sciences Research Corporation, 2002)^1^ and colour perception as assessed with the *Ishihara Test for Colour Blindness* (Ishihara, 1925). Performance on the length, size, orientation and picture naming (long version) subtests of the *Birmingham Object Recognition Battery* (BORB) (Riddoch & Humphreys, 1993) confirmed intact basic-level object recognition in all prosopagnosics. IQ, as measured with the *Raven Colored Progressive Matrices* (Raven, Raven, & Court, 1998) was also within the normal range for all prosopagnosics. None of the prosopagnosics reported any psychiatric or neurological problems.

**Fig. 3:**
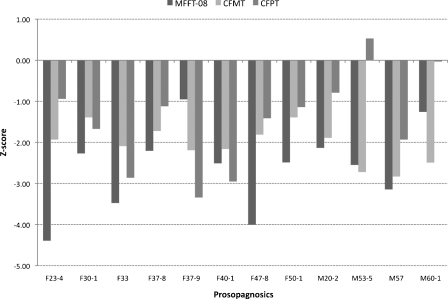
*Z*-scores of the 12 prosopagnosics on three tests of facial identity recognition, the MACCS Famous Face Test-08 (MFFT-08, Palermo et al., in preparation), the Cambridge Face Memory Test (CFMT, Duchaine & Nakayama, 2006) and the Cambridge Face Perception Test (CFPT, Duchaine et al., 2007). The prosopagnosics are labelled by sex and their age at completing the identity, and then expression, tests.

The presence of prosopagnosia – that is, the inability to reliably recognise facial identity – was determined using two tests of face memory and one of face perception. As can be seen in Fig. 3, the individuals in the prosopagnosic group performed poorly on these tests, and performed at least two standard deviations (SDs) below control norms on one or more tests. The *MACCS Famous Face Test 2008* (MFFT-08) assesses memory for famous faces that have generally been repeatedly seen over relatively long time periods (Palermo, Rivolta, Wilson, & Jeffery, in preparation). It contains 20 people famous to Australians and 20 that are not. On each trial: (a) a *face* is presented and participants judge whether it is familiar or not, (b) for the famous faces, they are asked to identify the *face* by providing its name or other specific autobiographical information, then (c) the famous person's *name* and relevant autobiographical information are presented, and participants report whether the famous *person* was actually known to them (any that are unknown are excluded from further analyses). The score on the MFFT-08 is the percentage of correctly recognised *faces* of *known* famous people. A sample of 39 control participants (26 females) aged between 19 and 72 years (*M* = 45.69, *SD* = 16.08) correctly recognised 74.17% (*SD* = 19.09) of known faces (Palermo et al., in preparation). Age-appropriate *z*-scores based on these control data were calculated for each prosopagnosic and vary from −0.95 to −4.39 (see Fig. 3).

The *Cambridge Face Memory Test* (CFMT, Duchaine & Nakayama, 2006) assesses face learning and memory. Participants learn six individuals (each from three different viewpoints), and then recognize the previously seen faces when shown in novel views and/or degraded by noise. Total scores on the upright CFMT were transformed to age-adjusted *z*-scores (using age-based norms reported in Bowles et al., 2009), with the prosopagnosics scoring between −1.39 and −2.83 below the Australian sample (Fig. 3).^2^

The *Cambridge Face Perception Test* (CFPT, Duchaine et al., 2007) requires participants to order a series of morphed faces in order of their likeness to a target face. *Z*-scores for upright faces, calculated using the age- and sex-based norms in Bowles et al. (2009), ranged from 0.53 to −3.34 (Fig. 3).^3^

All 12 prosopagnosics completed the tests of facial expression recognition and the expression composite effect, which were administered between 6 and 24 months after the identity tests (thus they were aged between 22 and 61 years; *M* = 41.67, *SD* = 12.87). Nine (*M* = 42.56, *SD* = 14.52) also completed the identity composite test (F37-8, F37-9, and F30-1 did not).

Some of these participants are also referred to in Bowles et al. (2009); Rivolta, Palermo, Schmalzl, and Coltheart (in press), and Palermo et al. (in preparation).

#### 2.1.2 Controls

For the three facial expression recognition tests, the expression composite effect and the CFMT, our controls comprised 17 participants without known brain injury (7 males), who were aged between 19 and 60 years (*M* = 38.94, *SD* = 14.42). They did not differ in age from the prosopagnosics, *t* < 1. (Note that we initially tested 21 control participants; however four were excluded, one for a CFMT *z*-score of −1.84, one for scoring below the ‘normal range’ cutoff given in the manual for the Ekman 60 Faces Test, and two for scoring below normal range cutoff values on both the Ekman 60 Faces and the Emotion Hexagon Test).

The identity composite effect was completed by a different group of control participants; *n* = 30 (7 males), aged between 23 and 62 years (*M* = 34.20, *SD* = 10.09). Once again, age did not differ from the prosopagnosics, *t*(37) = 1.96, *p* > .05.

### 2.2 Tests of facial expression recognition and social cognition

Recognition of basic and social expressions was assessed with three tests, following standard procedures. Results showed completely normal expression recognition in the CP group relative to the 17 controls (Table 1).

The *Ekman 60 Faces* (Young, Perrett, Calder, Sprengelmeyer, & Ekman, 2002) contains grayscale photographs of 10 individuals, each displaying one of six high-intensity prototypical basic emotions. Faces are presented, in random order, for 5 s each, and participants choose which emotion term (anger, disgust, fear, happiness, sadness, and surprise) best describes the facial expression shown. The number of correct responses out of 60 was computed. The mean performance of the group of prosopagnosics did not differ to that of our controls, *t* < 1 (Table 1). Importantly, this lack of difference cannot be attributed to a ceiling effect on the task (mean control accuracy was approximately 86%). We also confirmed that none of the individual prosopagnosics scored below cut-off scores that indicate the boundary between normal-range and impaired performance based on a large-N control sample as provided in the manual (i.e., 45 for ages 20–40, 43 for ages 41–60 and 41 for those aged 61–70 years).

The *Emotion Hexagon* Test (Young et al., 2002) consists of stimuli of graded difficulty, created by blending between two maximally confusable prototypical expressions (e.g., 90% happiness, 10% surprise; 70%, happiness, 30% surprise; 50% happiness, 50% surprise; 70% happiness, 30% surprise; 10% happiness, 90% surprise). Each of the thirty morphed faces is shown once in each of 5 blocks, for 5 s, in random order. Participants choose which emotion term (anger, disgust, fear, happiness, sadness, and surprise) best describes the facial expression. Total correct score out of 120 was computed. The prosopagnosic and control groups did not differ, *t* < 1 (Table 1), although note performance was close to ceiling on this task (control mean = 95%). We also compared individuals to large-N sample cut-offs in the manual (94 for ages 20–40, 92 for ages 41–60 and 90 for those aged 61–70 years). None of the prosopagnosics scored below cut-off.

The *Reading the Mind in the Eyes* (Revised) test (Baron-Cohen, Wheelwright, Hill, Raste, & Plumb, 2001) contains the eye-region of 36 faces displaying social emotions (e.g., flirtatious, pensive, sceptical). Participants are presented with four terms for each set of eyes and circle which word best describes what the person in the photograph was thinking or feeling. A page of word definitions is provided for reference. Adults with autism are impaired on this test, suggesting that this test taps subtle impairments in social intelligence (Baron-Cohen, Wheelwright, Hill, et al.). In the present study, the prosopagnosic and control groups did not differ, *t*(27) = 1.32, *p* > .2 (Table 1), with no ceiling effect (control mean = 87%) and in fact a small trend for the CP group to be *better* than controls. We further confirmed intact social expression perception in our CPs via comparison to published norms for a large-N sample from the general population (*n* = 122, mean age = 46.5 years, *SD* = 16.9; Baron-Cohen, Wheelwright, Hill, et al.): the large-N mean was 26.2 (*SD* = 3.6), and the lowest score here for a prosopagnosic was 25.

Participants also completed the *Autism Spectrum Quotient* (AQ) questionnaire (Baron-Cohen, Wheelwright, Skinner, & Clubley, 2001). None of the prosopagnosics scored 32 or above, which is indicative of an autism spectrum disorder (Baron-Cohen, Wheelwright, Skinner, et al.), and there was no difference between the prosopagnosics and controls, *t*(16.51, adjusted for unequal variance) = 1.40, *p* > .18 (Table 1). This is consistent with other recent work showing that CP can be clearly distinct from autism in both adults (Duchaine, Murray, Turner, White, & Garrido, 2009) and children (Wilson, Palermo, Schmalzl, & Brock, 2010).

### 2.3 Expression composite test

Each face displayed a composite of two emotions, one on the top half and a different one on the bottom half (e.g., fear on the top together with happiness on the bottom, see Fig. 1b). This test was essentially the same as that of Calder et al. (2000, Experiment 1), but with a different set of stimuli (because Calder et al. used the Ekman and Friesen Pictures of Facial Affect that were also contained in the Ekman 60 Faces Test that participants in our study had already completed). As in Calder et al., only expressions well recognised from the specific half were employed in that half: for the top half of the face emotions used were anger, fear, sadness; and for the bottom half of the face emotions used were happiness, disgust, surprise, of the same individual.

#### 2.3.1 Stimuli

The original whole faces (later used to make the composite stimuli) were grayscale photographs of four Caucasian individuals (two females), each displaying an angry, disgusted, fearful, sad, happy and surprised expression. The faces were sourced from the NimStim Face Stimulus Set (Models # 7 and 8) (Tottenham et al., 2009) and the Karolinska Directed Emotional Faces database (KDEF, Models # M09 and M17) (Lundqvist et al., 1998). A pilot study (*n* = 13) confirmed that the whole face expressions were well recognised (average recognition accuracy was 86.86%). Each of these faces was divided in half along the bridge of the nose to create the composite images. The pilot study also verified that participants were able to accurately recognise anger, fear, and sadness from the top halves presented alone (*M* = 80.45%) and happiness, disgust, and surprise from the bottom halves presented alone (*M* = 86.54%).

*Aligned* face composites were created by combining the top of an expression well-recognised from the top half of the face (i.e., anger, fear, sadness) with the bottom of an expression well-recognised from the bottom (i.e., happiness, disgust, surprise) of the same individual (see Fig. 1b). All nine possible combinations were formed for each of the four individuals, for a total of 36 aligned composites; there were thus, for example, 12 “happy”-target aligned trials, 4 where the happy expression was combined with anger on the top, 4 where happy was combined with fear on the top, and 4 where happy was combined with sadness. *Unaligned* composites were created by horizontally misaligning the top and bottom halves of the stimuli that were used to create the aligned composites so that the middle of the nose in the top segment was aligned with the edge of the face in the bottom segment. For half of the stimuli the top segment was shifted to the left of the bottom segment, while for the other half the top segment was shifted to the right. As neither the top nor bottom half of the unaligned images were centred on the screen, we therefore presented half of the aligned composites in the same position as the left segment of the aligned composites and half in the same position as the right segment of the unaligned composites.

#### 2.3.2 Procedure

The experiment commenced with a block of trials in which participants categorised the facial expression of each of the four *whole* faces posing each of the six facial expressions (24 trials) by pressing one of six labelled keys (anger, happiness, sadness, fear, surprise, disgust). This was then followed by one of two blocks: in one block participants were required to classify the facial expression depicted in the *top* half of the aligned and unaligned composites (as either anger, fear or sadness) via a key press, whereas in the other block, they were asked to classify the *bottom* half (as either happiness, surprise or disgust). Within each block, each aligned and unaligned composite was presented once, in a random order, for a total of 72 trials per block. Block order was counterbalanced between participants. Prior to commencing each block, participants classified an isolated top (or bottom) half of each individual displaying each expression (12 trials), and then 10 practice trials with the face halves combined into composites (half aligned, half unaligned). Each trial began with a fixation cross for 500 ms, followed by a 500 ms blank interval, and then the composite was presented until a response was made. An inter-trial-interval of 1000 ms preceded the commencement of the following trial. Participants were asked to respond as quickly and accurately as possible. Stimulus presentation was controlled by SuperLab (Cedrus Corp.) on a MacBook Pro with a 15-in. monitor, at a viewing distance of approximately 50 cm. Aligned composites were approximately 3.5 cm × 5.5 cm (4 × 6.5 degrees of visual angle) and unaligned were 5 cm × 5.5 cm (5.5 × 6.5 degrees of visual angle).

#### 2.3.3 Results

**Fig. 4:**
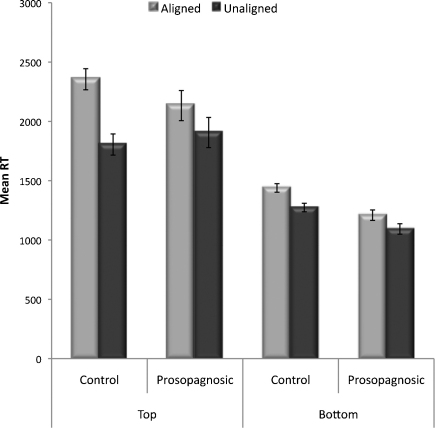
Mean RTs for the aligned vs. unaligned condition for controls and prosopagnosics, for expressions recognised from the top (anger, fear, sadness) and bottom (happiness, surprise, disgust) of a composite. Standard error bars are shown (adjusted for within-subject comparisons).

**Fig. 5:**
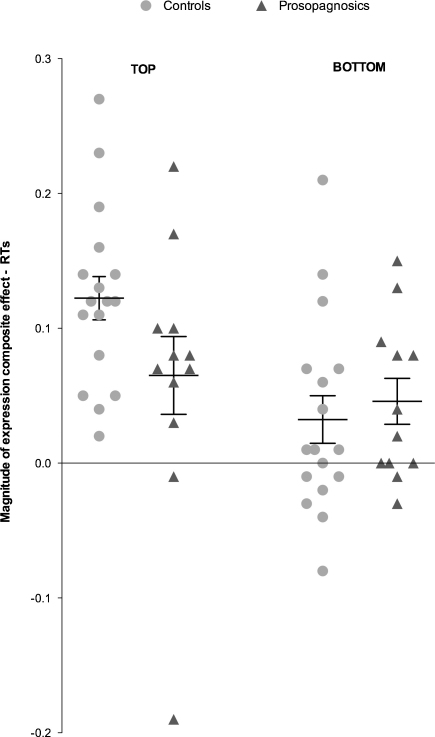
Normalised RT scores for expressions recognised from the top (anger, fear, sadness) and bottom (happiness, surprise, disgust) of a composite for each control and prosopagnosic. Means and SEMs displayed for each group.

Given the high accuracy rates on both our composite tasks, our analyses focus on response times (RTs) (percentage accuracy for the expression composite task is displayed in Table 2). Analysed RTs were for correct trials, excluding responses 3 SDs greater than the mean for each condition. Mean RTs were calculated for aligned and unaligned composites, for expressions judged from either the top (anger, fear, sadness) or the bottom (happiness, disgust, surprise) face half. Results (Figs. 4 and 5) showed a weaker expression composite effect in the CP group than in controls, particularly for top-half expression judgements, with even controls showing quite a small composite effect for bottom-half judgements. Supporting statistics were as follows.

A Group (prosopagnosics, controls) × Alignment (aligned, unaligned) × Half (top, bottom) ANOVA revealed main effects of Half, *F*(1,27) = 95.39, *p* < .001, $\eta_{p}^{2}=0.78\text{,}$ and Alignment, *F*(1,27) = 35.12, *p* < .001, $\eta_{p}^{2}=0.57\text{,}$ moderated by an Alignment × Half interaction, *F*(1,27) = 12.54, *p* < .001, $\eta_{p}^{2}=0\text{.32.}$ The interaction reflected a larger composite effect for top (aligned: *M* = 2244, SE = 134; unaligned: *M* = 1856, SE = 106) than bottom (aligned: *M* = 1324, SE = 72; unaligned: *M* = 1183, SE = 59) halves, although composite effects for both halves were statistically significant, *t*'s > 5.27, *p*'s < .001. The Group × Alignment × Half interaction approached significance, *F*(1,27) = 3.78, *p* = .06, $\eta_{p}^{2}=0.12\text{,}$ and most importantly, the Group × Alignment interaction was significant, *F*(1,27) = 4.33, *p* < .05, $\eta_{p}^{2}=0.14\text{.}$ Composite effects were evident for both groups, but significantly larger for controls, (aligned: *M* = 1897, SE = 123; unaligned: *M* = 1539, SE = 97), *t*(16) = 6.55, *p* < .001, than prosopagnosics (aligned: *M* = 1671, SE = 147; unaligned: *M* = 1499, SE = 116), *t*(11) = 2.35, *p* < .04 (see Fig. 4).

We also calculated the magnitude of the expression composite effect for each individual participant. To normalize for differences in baseline performance these were calculated as the relative difference in performance across conditions [(aligned − unaligned)/(aligned + misaligned)] (see Ramon, Busigny, & Rossion, 2010 for a similar procedure with an acquired prosopagnosic P.S.). Given these data were skewed we used a non-parametric test suited to small sample sizes, the Kolmogorov–Smirnov *Z* (Field, 2009). The magnitude of the composite effect for expressions displayed by the top half of the face was significantly weaker for prosopagnosics than controls, *z* = 1.43, *p* < .04, *r* = .27 (Fig. 5). There was no significant difference between the prosopagnosic and control groups for expressions displayed by the bottom half, *z* = .79, *p* = .55, *r* = .15.^4^

In sum, the group of CPs clearly show a composite effect for expression. However, the magnitude of the composite effect was significantly reduced for CPs compared to controls.

### 2.4 Identity composite test

#### 2.4.1 Stimuli and procedure

The identity composite effect stimuli were created by Le Grand, Mondloch, Maurer, and Brent (2004), who split photographs of unexpressive grayscale faces horizontally across the middle of the nose into top and bottom halves. The halves were recombined into different individuals that were *aligned* into an intact face, and spatially *unaligned*, with the top half of each face shifted to the left. The procedure was very similar to Le Grand et al. (2004, 2006). In brief, on each trial two face composites were sequentially presented (200 ms, with a 300 ms inter-stimulus interval) and participants judged whether the top halves were the same or different identity (the bottom halves were always different). A block of aligned composites (48 trials; half same top halves and half different top halves, randomly intermixed) was followed by a block of unaligned composites (also 48 trials) (note that block order does not affect performance, Le Grand et al., 2004). Four practice trials were presented prior to each block. Participants were asked to respond as quickly and accurately as possible. Stimulus presentation was controlled using SuperLab (Cedrus Corp.) on a Dell PC (19-in. monitor) or a MacBook Pro (15-in. monitor) from a distance of approximately 50 cm. Aligned composites were approximately 5 cm × 7.5 cm (5.5 × 8.5 degrees of visual angle) and unaligned were 8.5 cm × 7.5 cm (9.5 × 8.5 degrees of visual angle).

[Note that there has been recent discussion in the literature (e.g., Richler, Gauthier, Wenger, & Palmeri, 2008) about whether the traditional same-different version of the composite task, as used here, might tap a response bias to say “same” rather than the perceptual composite illusion. We chose to use the traditional version rather than the Gauthier-lab version involving additional conditions (e.g., Richler et al., 2008, 2011, in press) for several reasons. (a) A recent ERP study using the traditional conditions and monitoring for same-different changes as the behavioural task showed the composite effect was present early in visual processing (i.e., on the N170); this demonstrates a perceptual rather than decisional locus (Kuefner, Jacques, Prieto, & Rossion, 2010). (b) The particular Le Grand et al. (2004) stimulus set we used here has been confirmed to show the expected pattern of a large traditional composite effect upright in combination with no composite effect at all for the same faces inverted (Mondloch & Maurer, 2008); this demonstrates that the composite effect (misaligned − aligned difference for same trials) does not reflect a generalised bias to say ‘same’ more often to aligned trials. Also, (c) the Gauthier-lab version produces a large “composite effect” (i.e., congruency effect) for *inverted* faces (Richler et al., 2011), despite the lack of any perceptual illusion of integration of the two halves inverted (e.g., Young et al., 1987).]

#### 2.4.2 Results

**Fig. 6:**
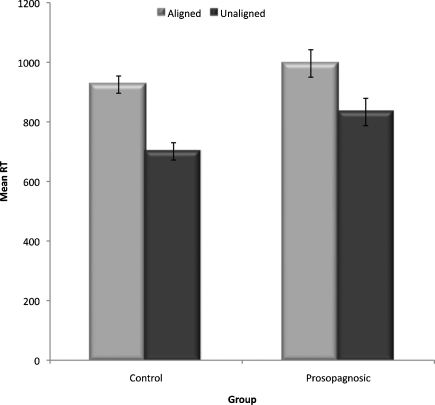
Mean RT for the aligned vs. unaligned condition for controls and prosopagnosics on the *same*-identity composite effect trials. Standard error bars are shown (adjusted for within-subject comparisons).

Mean RTs (for correct trials, excluding responses 3 SDs greater than the mean for each condition) were calculated for aligned and unaligned composites, for trials where the identities were the same, and ones where they were different. As is general practice, only the *same* trials were used to test for the presence of the composite effect (c.f., Le Grand et al., 2004; Robbins & McKone, 2007) (mean RTs for different trials and percent accuracy for same and different trials are shown in Table 3). A Group (prosopagnosics, controls) × Alignment (aligned, unaligned) ANOVA showed a composite effect, with slower RTs in the aligned (*M* = 960, SE = 39) than the unaligned (*M* = 767, SE = 26) condition, *F*(1,37) = 43.93, *p* < .0001, $\eta_{p}^{2}=0.54$ (see Fig. 6). There was no Group × Alignment interaction, *F*(1,37) = 1.06, *p* > .3. However, the most important result of the ANOVA was a marginal main effect of Group *F*(1,37) = 2.86, *p* = .099, $\eta_{p}^{2}=0.07\text{,}$ with prosopagnosics slower than controls, and an *a priori* comparison of the unaligned “baseline” condition showed that the prosopagnosics were significantly slower for unaligned trials than controls, *t*(37) = 2.50, *p* < .02. This means that the comparison of composite effects via the interaction in the ANOVA on raw scores is invalid, because it fails to take into account that controls, having a faster baseline RT, have theoretically *less* room to show a composite effect than CPs yet show a trend on the raw scores to showing a *larger* composite effect than CPs.

**Fig. 7:**
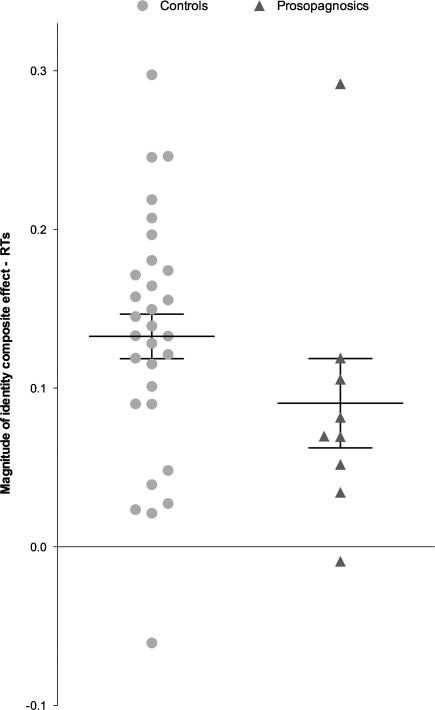
Normalised RT scores for the identity composite for each control and prosopagnosic. Means and SEMs displayed for each group.

Thus, as for the expression composite effect, the baseline-adjusted magnitude of the identity composite effect was calculated [(aligned − unaligned)/(aligned + misaligned)]. On this measure, the magnitude of the identity composite effect was significantly weaker for prosopagnosics than controls, *z* = 1.37, *p* < .05, *r* = .15 (Fig. 7). We also note that the baseline-adjusted composite effect was significant (i.e., greater than zero), for both controls, *t*(29) = 9.43, *p* < .001 and CPs, *t*(8) = 3.21, *p* < .02.

We also briefly examined the correlation, within the group of CPs (*n* = 9), between the size of their identity composite effect and the size of their expression composite effect for the top-half (both using baseline-adjusted scores). We used a non-parametric test, Kendall's tau (*τ*), which assesses the probability that the data are in the same order for the two variables, and has a range between −1 to 1 (StatSoft Inc, 2010). This correlation was small (although positive) and non-significant, *τ* = .17, *p* = .27, 1-tailed. Note, however, that we would not wish to rule out an association between these variables, given the low power with the small sample size. (Unfortunately, we were unable to conduct similar correlational analyses with the controls, because those who completed the identity composite task did not complete the other task.)

In sum, the results for identity are very similar to those for expression. That is, the identity composite effect for CPs is evident, but weaker.

## 3 Discussion

None of the 12 CPs we assessed was impaired on any of the three tests of basic and social facial expression recognition. These objective test scores agree with the CPs’ subjective reports of their everyday experience: all reported difficulty recognising facial identity (e.g., difficulty following films due to confusion about tracking the characters) but none reported difficulty recognising facial expression. Intact expression with impaired identity recognition is consistent with other studies (e.g., Duchaine et al., 2003; Humphreys et al., 2007), suggesting that this pattern may be common in CP. Note that we cannot rule out a subtle deficit in expression recognition emerging in CPs had they been tested on speeded expression recognition tasks. Importantly, however, the dissociation between expression and identity observed here cannot be attributed to differences in stimulus presentation duration. The expression tasks (Emotion Hexagon, Ekman 60, Reading the Mind in the Eyes) use long (>5 s) or unlimited presentation duration of faces. However, this is also true of the identity recognition tests used to diagnose the prosopagnosia: all our CPs were impaired either on a Famous Faces Test (unlimited stimulus duration) and/or the CFMT (6 s to learn each person; unlimited presentation duration at test). Thus, it is unlikely that a generic strategy which is easier to implement with long stimulus durations (e.g., sequential feature-by-feature analysis), can account for the dissociation between expression and identity recognition.

Our first question was to determine whether normal levels of facial expression recognition in our CPs were obtained via the use of normal perceptual mechanisms. Although our CPs did show holistic coding for expression, it was weaker than that seen for controls. This group of CPs also demonstrated normal facial expression recognition despite their weak holistic coding. Thus, they must be relying upon compensatory mechanisms that are either atypical (i.e., not used at all by controls) or typical but used to different degrees by controls (i.e., heavier use of non-holistic mechanisms in CP). This latter idea is plausible because there may be multiple effective expression recognition mechanisms (which may be less the case for identity recognition), some of which involve holistic coding but others which may involve focusing on single facial features (such as an upturned mouth for happiness; Ellison & Massaro, 1997) and/or “embodied cognition”, involving internal simulation of the emotion in somatosensory brain regions (Pitcher, Garrido, Walsh, & Duchaine, 2008). CPs could be using a subset of these other strategies, but relying on them more heavily than controls. Regardless of the precise mechanism relied upon by CPs, weak holistic coding implies that CPs were not recognising facial expressions in the same manner as controls.

The second question addressed by the current study was the locus of the dissociation between identity and expression recognition. The pattern of results for holistic coding of identity and expression were similar: CPs showed holistic coding, but it was weaker than in controls for both facial attributes. This result supports Model B (Fig. 2), in which there is an initial holistic processing stage that is common to both identity and expression. This stage of general holistic coding may be very early, with recent evidence for identity composite effects as early as 170 ms after stimulus onset (i.e., the face-sensitive N170 event-related potential, Jacques & Rossion, 2009, 2010; Letourneau & Mitchell, 2008). This early general holistic processing stage may also encompass other facial attributes in addition to identity and expression, given that composite effects are also seen for judgements of sex and attractiveness (Abbas & Duchaine, 2008; Baudouin & Humphreys, 2006; Zhao & Hayward, 2010).

The third question we examined was whether holistic processing for identity is functionally related to face identification ability. Consistent with proposals that holistic coding contributes to face recognition, we found that the identity composite effect was weaker in CPs (who by definition are very poor at recognising face identity) than in controls (who we confirmed were normal at recognising face identity). Weak holistic coding of identity is also seen in other groups of individuals with developmental disorders affecting face perception. A group of 12 individuals who were deprived of early patterned visual input by bilateral congenital cataracts for 3–6 months after birth displayed a significantly smaller composite effect when assessed with essentially the same composite test as used here (Le Grand et al., 2004). Group studies of adolescents with autism, a neurodevelopmental disorder in which individuals often display face identity and expression recognition impairments (Sasson, 2006; Wilson et al., 2010), also reveal impaired holistic coding, as measured with the composite effect (Gauthier, Klaiman, & Schultz, 2009; Teunisse & de Gelder, 2003). Our claim that holistic coding contributes to identity recognition is not supported by Konar et al.’s (2010) study of individual differences across the normal population. However, as noted earlier, associations may have been masked by the use of a face matching, rather than recognition memory, task. Richler et al. (in press) did not observe a relationship between face recognition ability on the CFMT and strength of the identity composite effect assessed via the traditional same-different version of the composite task (but note that *n* = 34). They did find a relationship between CFMT scores and the Gauthier-lab version (‘congruency effect’; but see earlier note for discussion of limitations of this version). As such, the present study is the first to show a relationship between face recognition ability and strength of the standard composite effect. This leaves open the possibility that the relationship only becomes apparent with a wide range of CFMT scores (available when including CPs) and is either absent or more difficult to observe in the smaller range afforded by the normal population.

We note that the holistic processing deficits seen in our study of CPs are milder than those reported in acquired prosopagnosia. In acquired prosopagnosia, case studies have reported a complete lack of holistic coding, for both expression (Case H.J.A., Baudouin & Humphreys, 2006) and identity (Case P.S., Ramon et al., 2010). The difference in results may be related to severity of prosopagnosia. That is, the acquired prosopagnosics tested to date recognise few if any faces (H.J.A. and P.S. identified less than 1% of famous faces, Baudouin & Humphreys, 2006; Rossion et al., 2003), and display negligible levels of holistic coding, while CPs who can typically recognise a modest proportion of faces (e.g., the CPs in our present study recognised 33% of famous faces on average), have weak but not completely absent holistic coding. Our finding of weak, but not absent, holistic coding in CP may be consistent with suggestions that CPs are at the lower end of a continuum with normals, of both holistic processing, and of face identity recognition abilities (see Bowles et al., 2009 for discussion).

It is also important to note that we do not wish to argue that weak holistic coding is the only, or even primary, deficit in CP. First, our CPs did show holistic coding, albeit weaker than controls on average. Second, Figs. 5 and 7 suggest there may be heterogeneity between individuals: some individual CPs in the current study appear to display normal levels of holistic coding of expression or identity. For identity, this has also been reported in previous studies (Le Grand et al., 2006; Schmalzl et al., 2008), although we note that composite findings from *individual* participants cannot necessarily be taken as reliable from a single composite test, given that the internal reliability of this task is generally not high (e.g., split-half reliability = .65 in Zhu et al., 2010), and thus evidence of normal holistic processing in individual CPs would thus ideally require confirmation from two or more versions of an identity (or expression) composite task.

To summarise, our CPs as a group displayed normal facial expression recognition, together with impaired facial identity recognition, and weakened holistic processing of both expression and identity. The expression findings suggest an increased use of compensatory non-holistic strategies for expression recognition. The identity findings support a view that holistic coding is functionally involved in face identification. Finally, the findings involving expression and identity in concert, are consistent with a model proposing a general, early, holistic coding stage for multiple facial attributes.

## Funding sources

This research was supported by funding from Macquarie University and the Australian National University [RP], Australian Research Council's *Discovery Projects* funding scheme (project numbers: DP110100850) [EM and RP] and DP0984558 [EM]) and the MRC (grant code 513111 MC_US_A060_0017 [AJC]).

These funding sources played no role in study design; in the collection, analysis, and interpretation of data; in the writing of the report; or in the decision to submit the paper for publication.

## Figures and Tables

**Table 1:** Scores on the *Ekman 60 Faces* and *Emotion Hexagon* tests (Young et al., 2002), the *Reading the Mind in the Eyes* test (Baron-Cohen et al., 2001), and the *Autism Spectrum Quotient* (AQ) (1) for each of the prosopagnosics, and means and standard deviations for the prosopagnosic (*n* = 12) and control (n = 17) groups.

	Ekman 60 Faces (/60)	Emotion Hexagon (/120)	Reading the Mind in the Eyes (/36)	Autism Spectrum Quotient (AQ)
*Prosopagnosics*				
F23-4	55	119	33	16
F30-1	53	120	28	11
F33	51	120	31	11
F37-8	52	115	28	3
F37-9	50	115	32	11
F40-1	53	112	28	14
F47-8	55	113	27	30
F50-1	52	111	29	22
M20-2	54	110	25	23
M53-5	49	102	29	22
M57	49	111	28	20
M60-1	58	118	33	28
Mean (SD) (*n* = 12)	52.58 (2.68)	113.83 (5.20)	29.25 (2.49)	17.58 (7.95)
*Controls* (*n* = 17)				
Mean (SD)	52.00 (4.03)	114.29 (3.41)	27.94 (2.73)	14.00 (4.74)

**Table 2:** Percent accuracy for the aligned vs. unaligned conditions for controls and prosopagnosics, for expressions recognised from the top (anger, fear, sadness) and bottom (happiness, surprise, disgust) of a composite (standard errors, adjusted for within-subject comparisons in parentheses).

	Top		Bottom	
	Aligned	Unaligned	Aligned	Unaligned
Controls	85.65 (1.38)	87.82 (1.38)	92.35 (1.46)	95.35 (1.46)
Prosopagnosics	82.83 (2.56)	84.25 (2.56)	96.33 (1.12)	96.92 (1.12)

**Table 3:** Percent accuracy for *same*-identity trials and percent accuracy and mean RTs for *different*-identity trials (standard errors, adjusted for within-subject comparisons in parentheses).

	*Same*-identity trials		*Different*-identity trials			
	Percent accuracy		Percent accuracy		Mean RTs (ms)^a^	
	Aligned	Unaligned	Aligned	Unaligned	Aligned	Unaligned
Controls	80.97 (2.74)	91.37 (2.74)	81.53 (2.98)	80.27 (2.98)	940 (27)	806 (27)
Prosopagnosics	80.56 (4.07)	92.60 (4.07)	80.56 (2.84)	79.63 (2.84)	1043 (45)	928 (45)

a One prosopagnosic (M53-5) was excluded as his mean RTs were consistently longer than the other participants: 2690 ms (aligned) and 5706 ms (unaligned).

## Footnotes

1 The FACT was not administered to F47-8 and M57.

2 The CFMT *z*-score (−1.39) for F30-1 likely overestimates her face recognition skills as it was the second time she completed the test (the initial score six weeks prior was not recorded due to computer malfunction) and controls, on average, improve 6.3 percentage points when re-tested approximately 6 months later (Wilmer et al., 2010).

3 For the four CPs with normal-looking CFPT performance (F23-4, M53-5, M20-2, M60-1), holistic coding strength was not consistently greater, or lower, than that of the other prosopagnosics. Thus, we opted to include all 12 CPs whose performance we assessed, on the basis that they are representative of the typical CP population.

4 Given that happy faces are typically recognized more accurately/rapidly than other expressions, we also analysed the bottom-half-target condition leaving out the happy-target trials. The results did not change: there was still no difference in baseline adjusted composite effect scores for CPs and controls, *z* = .57, *p* = .90.
